# Salt induction and activation of MtlD, the key enzyme in the synthesis of the compatible solute mannitol in *Acinetobacter baumannii*

**DOI:** 10.1002/mbo3.614

**Published:** 2018-03-24

**Authors:** Sabine Zeidler, Josephine Hubloher, Patricia König, Ngoc Dinh Ngu, Anica Scholz, Beate Averhoff, Volker Müller

**Affiliations:** ^1^ Molecular Microbiology & Bioenergetics Institute of Molecular Biosciences Johann Wolfgang Goethe University Frankfurt am Main Frankfurt Germany

**Keywords:** *Acinetobacter baumannii*, desiccation, enzyme activity, gene expression, mannitol, regulation

## Abstract

Mannitol is the major compatible solute, next to glutamate, synthesized by the opportunistic human pathogen *Acinetobacter baumannii* under low water activities. The key enzyme for mannitol biosynthesis, MtlD, was identified. MtlD is highly similar to the bifunctional mannitol‐1‐phosphate dehydrogenase/phosphatase from *Acinetobacter baylyi*. After deletion of the *mtlD* gene from *A. baumannii* ATCC 19606^T^ cells no longer accumulated mannitol and growth was completely impaired at high salt. Addition of glycine betaine restored growth, demonstrating that mannitol is an important compatible solute in the human pathogen. MtlD was heterologously produced and purified. Enzyme activity was strictly salt dependent. Highest stimulation was reached at 600 mmol/L NaCl. Addition of different sodium as well as potassium salts restored activity, with highest stimulations up to 41 U/mg protein by sodium glutamate. In contrast, an increase in osmolarity by addition of sugars did not restore activity. Regulation of mannitol synthesis was also assayed at the transcriptional level. Reporter gene assays revealed that expression of *mtlD* is strongly dependent on high osmolarity, not discriminating between different salts or sugars. The presence of glycine betaine or its precursor choline repressed promoter activation. These data indicate a dual regulation of mannitol production in *A. baumannii*, at the transcriptional and the enzymatic level, depending on high osmolarity.

## 1 INTRODUCTION

*Acinetobacter* species cope with low water activities by the accumulation of compatible solutes. The nonpathogen *A. baylyi* synthesizes glutamate and mannitol de novo in response to increasing osmolarities of the medium (Sand, Mingote, Santos, Müller, & Averhoff, 2013). If glycine betaine or choline are present they are taken up from the environment (Sand et al., 2011). Uptake of compatible solutes is energetically favored over de novo synthesis (Oren, 1999) and, thus, synthesis of mannitol or glutamate is turned off in the presence of glycine betaine or choline (Sand et al., 2013). The latter is taken up by *A. baylyi* and oxidized to glycine betaine (Scholz, Stahl, de Berardinis, Müller, & Averhoff, 2015). The closely related opportunistic pathogen *A. baumannii* has become a major threat in health care institutions worldwide. Its increasing success is caused by acquiring resistances to different antibiotics, its enormous metabolic potential that allows it to adapt to the host environment, the ability to adhere to biotic and abiotic surfaces, and its desiccation resistance which allows the cells to survive for weeks and even months on inanimate surfaces (Averhoff, 2015; Dijkshoorn, Nemec, & Seifert, 2007; Lee et al., 2017; Peleg et al., 2012; Roca, Espinal, Vila‐Farrés, & Vila, 2012). Desiccation resistance of *A*. *baumannii* favors survival and spread of the bacterium in the health care environment (Dijkshoorn et al., 2007). The enormous desiccation resistance is unusual for a gram‐negative bacterium and its molecular basis is enigmatic (Jawad, Heritage, Snelling, Gascoyne‐Binzi, & Hawkey, 1996). Changes in surface structures, lipid composition, biofilm formation, and composition of osmolytes inside the cell may contribute to this phenotype (Boll et al., 2015; Espinal, Martí, & Vila, 2012; Gayoso et al., 2014). We recently identified mannitol and glutamate as major compatible solutes of *A. baumannii* grown under low water activities (Zeidler et al., 2017). In addition, minor amounts of trehalose are synthesized. Mannitol is the most abundant sugar alcohol in nature. It serves as carbon and energy source or radical scavenger and in plants and fungi it is well known as compatible solute (Chaturvedi, Wong, & Newman, 1996; Stoop, Williamson, & Pharr, 1996). In bacteria, only *Pseudomonas putida* (Kets, Galinski, de Wit, de Bont, & Heipieper, 1996), *A. baylyi* (Sand et al., 2013), and *Gluconobacter oxydans* (Zahid, Schweiger, Galinski, & Deppenmeier, 2015) have been reported to accumulate the compatible solute mannitol. The biosynthetic route for the production of mannitol as compatible solute in *A. baylyi* was unraveled only recently. NMR analysis revealed Mtl‐1‐P as intermediate, that was further dephosphorylated leading to mannitol (Sand et al., 2013). Both reactions are catalyzed by MtlD, a novel bifunctional mannitol‐1‐phosphate dehydrogenase/phosphatase (Sand et al., 2015). Apparently, the enzyme is widespread in members of the genus *Acinetobacter* but restricted to the genus. Here, we have addressed mannitol biosynthesis and its regulation in the opportunistic human pathogen *A. baumannii* ATCC 19606^T^.

## 2 MATERIALS AND METHODS

### 2.1 *Bacterial strains and culture conditions*

*A. baumannii* strain ATCC 19606^T^ was grown at 37°C and 130 rpm in minimal medium consisting of 50 mmol/L phosphate buffer (pH = 6.8), different mineral salts (1 g/L NH_4_Cl, 580 mg/L MgSO_4_ × 7 H_2_O, 100 mg/L KNO_3_, 67 mg/L CaCl_2_ × 2 H_2_O, 2 mg/L (NH_4_)_6_MoO_24_ × 4 H_2_O), 1 ml of the trace element solution SL9 (12.8 g/L titriplex, 2 g/L FeSO_4_ × 7 H_2_O, 190 mg/L CoCl_2_ × 6 H_2_O, 122 mg/L MnCl_2_ × 4 H_2_O, 70 mg/L ZnCl_2_, 36 mg/L MoNa_2_O_4_ × 2 H_2_O, 24 mg/L NiCl_2_ × 6 H_2_O, 6 mg/L H_3_BO_3_, 2 mg/L CuCl_2_ × H_2_O, modified after Tschech and Pfennig (1984)) and 20 mmol/L sodium succinate as carbon source.

*E. coli* BL21 (DE3) was cultured at 37°C in LB medium (Bertani, 1951). Antibiotics were added when appropriate (20 μg kanamycin/ml).

### 2.2 Markerless mutagenesis

A markerless *mtlD* deletion mutant of *A. baumannii* ATCC 19606^T^ was generated as described by Stahl, Bergmann, Göttig, Ebersberger, and Averhoff (2015). All primers used are listed in the Supporting Information (S. 1). 1500 bp up‐ and downstream of the *mtlD* gene (HMPREF0010_00722) was amplified from genomic DNA using the primer pair mtlD_up_fwd and mtlD_up_rev and the primer pair mtlD_down_fwd and mtlD_down_rev. The upstream DNA fragment spans the first 55 bp of *mtlD* and the downstream fragment spans the last 54 bp of *mtlD*. The PCR fragments were cloned into pBIISK_sacB/kanR using PstI, BamHI, and NotI. The resulting plasmid pBIISK_sacB/kanR_mtlD‐updown was used for transformation of electrocompetent *A. baumannii* ATCC 19606^T^. Transformants were selected on LB‐agar + 50 μg/ml kanamycin and integration of the plasmid in the target locus *via* single homologous recombination was verified by PCR using the primer pairs mtlD_ctr_fwd + mtlD_down_rev and mtlD_up_fwd + mtlD_ctr_rev. Integrants were grown overnight in LB + 10% sucrose and subsequently plated on LB‐agar + 10% sucrose for counterselection of clones that lost the plasmid. Single colonies that had lost their ability to grow on kanamycin were verified by PCR with the primers mtlD_ctr_up + mtlD_ctr_down. The deletion of the target gene was confirmed by sequencing of the PCR product.

### 2.3 Solutes extraction and quantification

Analysis of mannitol was performed as described previously (Zeidler et al., 2017). Briefly, a modified Bligh‐and‐Dyer method (Bligh & Dyer, 1959; Galinski & Herzog, 1990) was used for extraction of freeze‐dried cell pellets. A quantity of 570 μl of extraction solution (methanol/chloroform/H_2_O_deion_ 10:5:4) were added to 15‐20 mg of lyophilized cells, vigorously vortexed and shaken for 5 min. A quantity of 170 μl chloroform and 170 μl H_2_O_deion_ were added prior to mixing the sample again (10 min). Phases were separated by centrifugation, the upper aqueous phase was dried in a vacuum concentrator and the residue was dissolved in 500 μl H_2_O_deion_.

Mannitol was determined using a DIONEX HPLC system equipped with a ligand exchange column (HyperREZ XP Carbohydrate Ca^2+^, Thermo Scientific) with water as eluent. Chromatography was carried out with a flow rate of 0.6 ml/min at 80°C. A refractive index detector was coupled to the system for detection.

### 2.4 Cloning, enzyme production, and purification

For overproduction of MtlD, the *mtlD* gene (HMPREF0010_00722) was amplified from chromosomal DNA using the primers mtlD_fwd and mtlD_rev (Supporting Information S. 1). The PCR product was cloned into the expression vector pET21a using the restriction enzymes NdeI and NotI, yielding the plasmid pET21a_mtlD. Gene expression in *E. coli* BL21 (DE3) was induced by addition of IPTG to a final concentration of 1 mmol/L after the cells reached an OD_600_ of 0.4. After incubation at 16°C over night the cells were harvested at 7,000xg for 10 min at 4°C and washed and resuspended in 50 mmol/L NaH_2_PO_4_ × 2 H_2_O, 300 mmol/L NaCl, and 20 mmol/L imidazole (pH 7.5). The cells were disrupted *via* French Press (two times, 1,000 psi) and the cell debris was removed by centrifugation at 17,000xg at 4°C for 15 min. Prior to the column loading, the supernatant was incubated with 5 ml of Ni‐NTA material for 1 hr at 4°C. In order to purify MtlD, the column was washed with 50 ml of washing buffer 2 (50 mmol/L NaH_2_PO_4_ × 2 H_2_O, 300 mmol/L NaCl, 75 mmol/L imidazole, pH 7.5) before the protein was eluted with 10 ml of elution buffer (50 mmol/L NaH_2_PO_4_ × 2 H_2_O, 300 mmol/L NaCl, 300 mmol/L imidazole, pH 7.5). The protein concentration was determined by Bradford (1976). Proteins were separated on a 12.5% sodium dodecyl sulfate polyacrylamide gel electrophoresis (SDS‐PAGE) according to Laemmli (1970) and stained with Coomassie (0.265 g Serva Blue R‐250, 50 ml methanol, 50 ml glacial acetic acid, H_2_O_deion_ ad. 500 ml).

### 2.5 Enzyme assay

MtlD activity was determined by a spectrophotometric assay as described before (Sand et al., 2013), following the oxidation of NADPH at 340 nm. By default, the reaction was carried out at 37°C in a total volume of 1 ml, containing 50 mmol/L MOPS, pH 7.0, 50 mmol/L fructose‐6‐phosphate and 10 μg MtlD. The reaction was started by addition of 0.4 mmol/L NADPH.

### 2.6 Size exclusion chromatography

For size exclusion chromatography a Superdex 200 10/300 GL column (bed volume 24 ml) was used. The run was performed at a flow rate of 0.2 ml/min in a buffer containing 20 mmol/L HEPES, 300 mmol/L NaCl, 5 mmol/L β‐mercaptoethanol pH 7.5 in an ÄKTAprime plus (Amersham Biosciences). A quantity of 100 μg protein were applied to the column. The following proteins were used for calibration: aprotinine (6.5 kDa), carboanhydrase (29 kDa), ovalbumine (44 kDa), conalbumine (75 kDa), aldolase (158 kDa), ferritine (440 kDa), thyroglobuline (669 kDa).

### 2.7 Construction of the reporter gene plasmid

The promoterless β‐glucuronidase gene (*gusA*) was used as reporter gene. 699 bp upstream of the *mtlD* gene were fused with the *gusA* gene. Therefore, the upstream region of *mtlD* was amplified using the primers mtlD_up_gusA_fwd and mtlD_up_gusA_rev (Supporting Information S. 1). The pIM1440 plasmid containing *gusA* was used as backbone (Murin, Segal, Bryksin, & Matsumura, 2012). The plasmid and the PCR fragment were digested with XbaI and NcoI and then ligated. Thereby, the T5‐Promotor and *lac* operator in front of *gusA* were replaced by the upstream region of *mtlD*. *A. baumannii* ATCC 19606^T^ was transformed with the reporter gene plasmid *via* electroporation (Stahl et al., 2015).

### 2.8 Reporter gene assay

Cells containing the pBAV1k_mtlD‐up‐gusA plasmid were grown in minimal medium. In the exponential growth phase (2–2.5 hr, OD_600_ 0.4–0.5) the extracellular osmolarity was increased as described in the results. To monitor *mtlD* promoter activity, samples (500 μl) were taken and analyzed according to Zhang and Bremer (1995). Briefly, cells were resuspended and thereby disrupted in 300 μl permeabilization solution (50 mmol/L Na_2_HPO_4_, 20 mmol/L KCl, 2 mmol/L MgSO_4_, 2.2 mmol/L CTAB, 1 mmol/L sodium deoxycholate, and 0.54% (v/v) β‐mercaptoethanol), 600 μl of prewarmed (37°C) substrate solution (60 mmol/L Na_2_HPO_4_, 40 mmol/L NaH_2_PO_4_, 0.27% (v/v) β‐mercaptoethanol, 2.77 mmol/L *p*‐nitrophenyl‐β‐D‐glucuronide) was added to start the β‐glucuronidase reaction. The reaction was stopped by adding 700 μl 1 mol/L Na_2_CO_3_. Miller Units were calculated according to Miller (1972).

## 3 RESULTS

### 3.1 Genomic organization of *mtlD of A. baumannii* and properties of the deduced gene product

**Figure 1:**
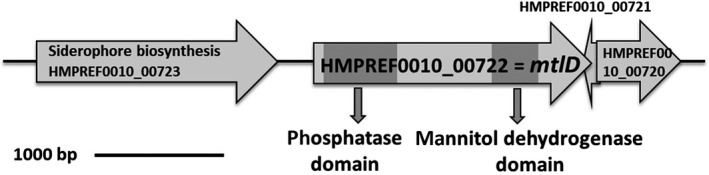
Genetic organization of *mtlD* in *A. baumannii* ATCC 19606^T^ (HMPREF0010_00722). The two functional domains are indicated

*mtlD* of *A. baumannii* (Figure 1) is 2148 bp long. 332 bp upstream of *mtlD*, in the same direction of transcription, a gene encoding a potential siderophore biosynthesis protein was detected. Downstream of *mtlD* are two genes encoding conserved hypothetical proteins, one is divergently transcribed from *mtlD* with an overlap of 23 bp and the other is in the same direction of transcription (81 bp apart). The GC content of *mtlD* is 36.5% which is in the same range as the overall GC content of the genome of *A. baumannii* (39%–47%) (Baumann, Doudorof, & Stanier, 1968). *mtlD* codes for a hydrophilic protein of 82 kDa. It is 68% identical to MtlD of *A baylyi*. MtlD of *A. baylyi* is a bifunctional enzyme having a dehydrogenase and a phosphatase domain (Sand et al., 2015). The dehydrogenase and phosphatase domains are 77 and 64% identical to the analogous domains in MtlD of *A. baylyi*, indicating that MtlD of *A. baumannii* is also a bifunctional mannitol‐1‐phosphate dehydrogenase/phosphatase.

### 3.2 The gene *mtlD* is essential for mannitol biosynthesis

**Figure 2:**
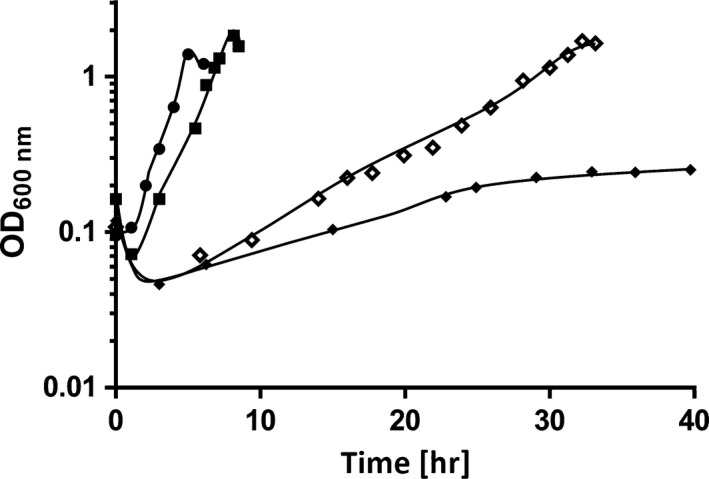
Growth of *A. baumannii* Δ*mtlD* under osmotic stress. *A. baumannii* Δ*mtlD* was grown in mineral medium (●), in mineral medium with 500 mmol/L NaCl (♦) or with 500 mmol/L NaCl and the addition of 1 mM glycine betaine (■). Growth of wild type at 500 mmol/L NaCl is plotted for comparison (◊). One representative experiment out of at least three independent biological replicates is shown

To address the hypothesis that MtlD of *A. baumannii* catalyzes mannitol formation, a deletion mutant was generated as described in Materials and Methods. At low salt the Δ*mtlD* mutant exhibited a growth phenotype comparable to *A. baumannii* wild‐type cells. However, at high salt the Δ*mtlD* mutant showed almost no growth (Figure 2). The wild‐type growth phenotype at high salt concentrations could be completely restored by addition of 1 mmol/L glycine betaine to the medium (Figure 2), which is known to serve as a compatible solute in many bacteria including *Acinetobacter* species, indicating that mannitol is an essential compatible solute that cannot be substituted by the other endogenous solutes glutamate or trehalose (Sand et al., 2011; Zeidler et al., 2017). To prove that MtlD is indeed essential for mannitol biosynthesis, we analyzed the intracellular mannitol pool at a lower salt concentration, where growth of the Δ*mtlD* mutant was still possible. At 300 mmol/L NaCl, growth occurred up to an OD_600_ of 1.5 with a growth rate of 0.37 ± 0.02 hr^−1^ (data not shown). Indeed, mannitol could not be detected in the mutant cells under these conditions, whereas the wild type produced 0.2 μmol/mg protein (Zeidler et al., 2017).

### 3.3 Overproduction and purification of *MtlD* from *A. baumannii* ATCC 19606^T^

**Figure 3:**
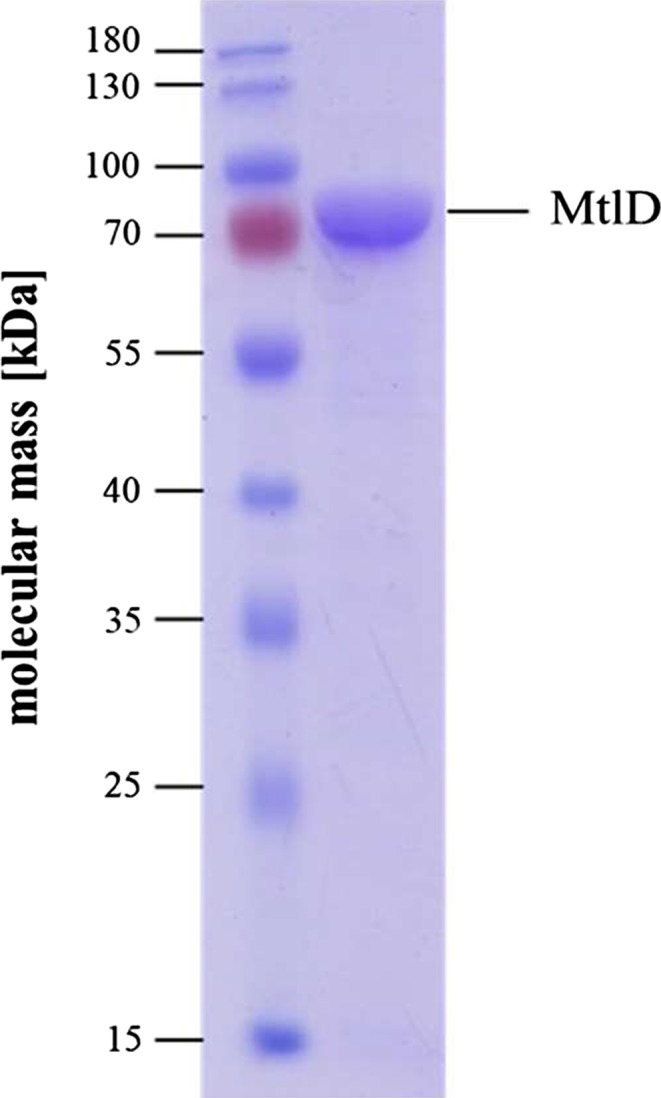
Purified mannitol dehydrogenase MtlD of *A. baumannii*. The enzyme was purified by Ni‐NTA (elution at 300 mmol/L imidazole) and analyzed on a 12.5% SDS gel. A quantity of 5 μg of protein was applied to the gel and stained with Coomassie Brilliant Blue R‐250

To address the catalytic activity of MtlD, the encoding gene was cloned into the expression vector pET21a and expressed in *E. coli*. The coding sequence was fused in frame to a His‐tag‐encoding sequence at its 3’ terminus. The fusion protein was purified *via* affinity chromatography on a Ni‐NTA resin. As shown in Figure 3, MtlD had an apparent molecular mass of 82 kDa which corresponds to its deduced molecular mass and was purified in one step to apparent homogeneity. This result was confirmed by size exclusion chromatography, where one predominant peak occurred, corresponding to a molecular mass of 87 kDa.

### 3.4 Properties of MtlD

**Figure 4:**
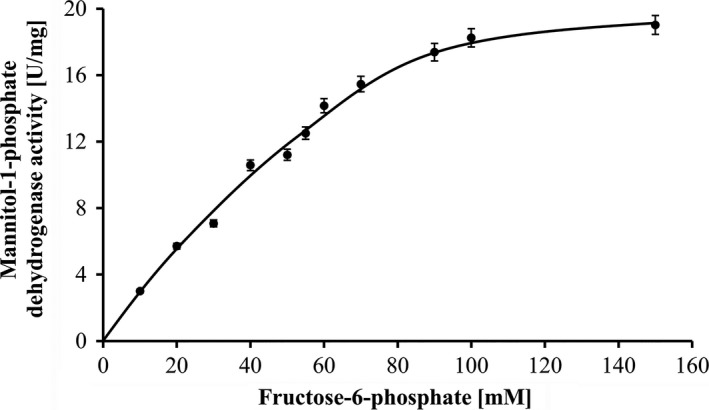
Substrate dependence of MtlD activity. The enzyme assay (1 ml) was performed at 37°C in 50 mmol/L MOPS, pH 7.0, with 0.6 mol/L NaCl, 10 μg of MtlD and increasing concentrations of the substrate fructose‐6‐phosphate. The mixture was preincubated at 37°C for 5 min before the reaction was started by the addition of 0.4 mmol/L NADPH. The activity is given in U/mg protein. Each measurement was performed as technical duplicate. Standard deviation of three biological replicates is given as error bars

The purified enzyme catalyzed fructose‐6‐phosphate‐dependent oxidation of NADPH with a specific activity of 20 U/mg protein in the presence of 600 mmol/L NaCl; this is 20% of the activity of MtlD from *A. baylyi* (Sand et al., 2013). NADH was also used as reductant, but the activity was fivefold lower. Activity increased with increasing fructose‐6‐phosphate concentrations obeying a Michaelis‐Menten kinetic (Figure 4). A plateau was reached at 100 mmol/L. MtlD of *A. baumannii* had a rather low affinity for its substrate fructose‐6‐phosphate with an apparent K_M_ of 55 mmol/L. The pH optimum was rather broad with a maximum at pH 7, however, only 12% of the maximal activity were detected at pH 5 and 10. The temperature optimum was at 37°C, with 40% activity at 20 and 45°C (data not shown).

### 3.5 Activity of MtlD is strictly salt dependent

**Figure 5:**
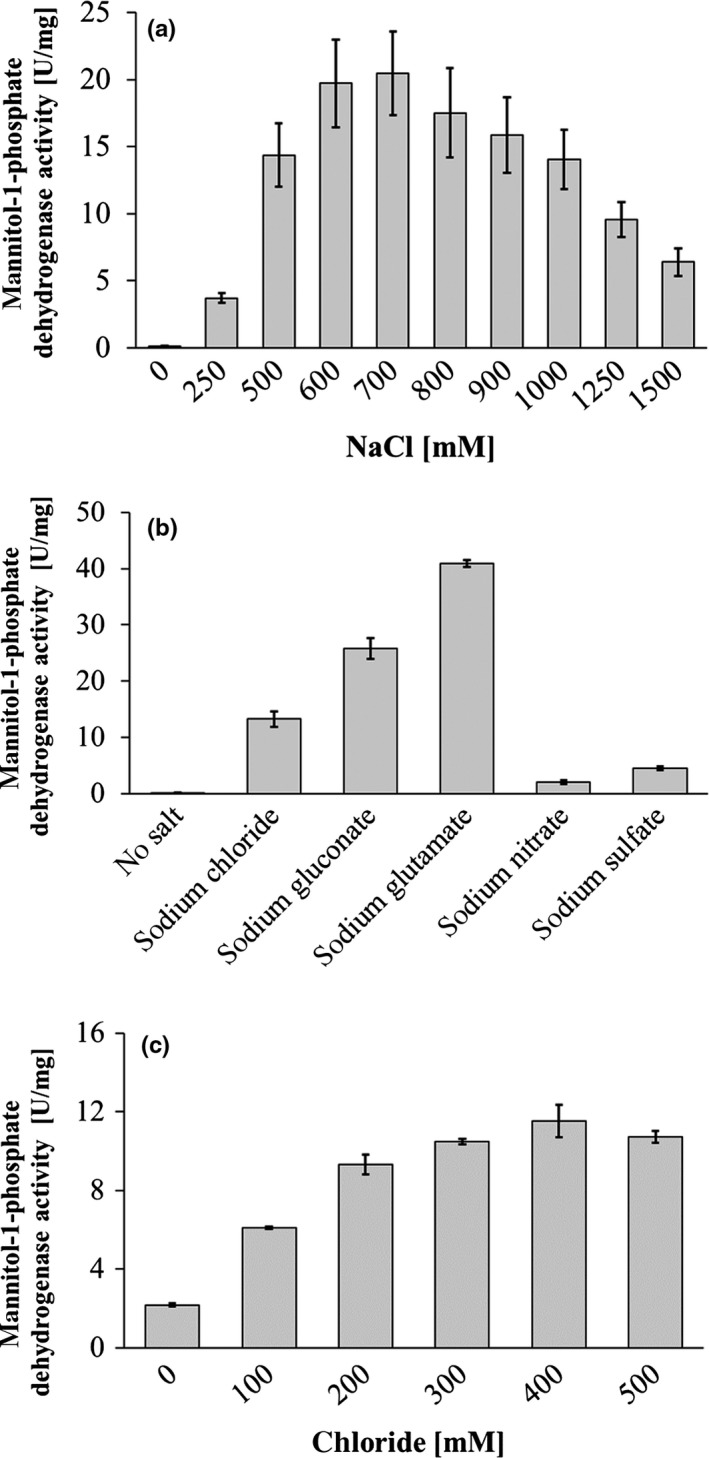
Salt dependence of MtlD activity. The enzyme assay (1 ml) was performed at 37°C in 50 mmol/L MOPS, pH 7.0, with 50 mmol/L fructose‐6‐phosphate, varying salt concentrations and 10 μg of MtlD. The buffer was preincubated at 37°C for 5 min before the reaction was started by the addition of 0.4 mmol/L NADPH. The activity is given in U/mg protein. Each measurement was performed as technical duplicate. Standard deviation of three biological replicates is given as error bars. (a): Dependence on NaCl concentrations. (b): Dependence on different sodium salts (1 mol/L each). (c): Chloride dependence of MtlD activity. Chloride concentrations were adjusted by addition of NaCl, the overall salt concentration was kept constant at 0.5 mol/L by appropriate addition of sodium gluconate

The enzymatic assays described before were performed in the presence of 600 mmol/L NaCl since MtlD of *A. baylyi* was described to require NaCl for activity (Sand et al., 2013). To address and quantify a potential salt dependence of MtlD of *A. baumannii*, the enzyme was incubated in buffer containing different amounts of NaCl. As can be seen in Figure 5a, there was no activity in the absence of NaCl. Activity was restored by NaCl in a concentration‐dependent manner. Maximal activity was detected in buffer with 600–700 mmol/L NaCl. Higher NaCl concentration led to a decline in MtlD activity. At 1.5 mol/L NaCl, activity was only 24%. These data provide clear evidence that MtlD activity is strictly salinity dependent.

When used at 1 mol/L concentration, sodium nitrate and sodium sulfate stimulated only weakly. However, sodium gluconate and sodium glutamate were superior over NaCl (Figure 5b). The concentrations of the different salts required for maximal activity were different: with sodium glutamate, maximal activity (200% compared to maximum with NaCl) was obtained at 0.8–1.2 mol/L, with sodium gluconate at 1.5 mol/L (135%), with Na_2_SO_4_ at 1 mol/L (35%), and with NaNO_3_ at 0.6 mol/L (25%).

To determine whether the stimulatory effect of the sodium salts is due to the presence of sodium ions, MtlD activities in the presence of different potassium salts were analyzed (data not shown). KCl stimulated activity as well with a maximum at 800 mmol/L KCl (13 U/mg protein), at 1.5 mol/L KCl activity was still 52%. Potassium gluconate and potassium glutamate also stimulated activity with a maximum at 1.4 mol/L (28 U/mg protein) and at 1.25 mol/L (41 U/mg protein), respectively. Interestingly, also MgCl_2_ stimulated activity, but maximal activity was detected already with 0.1 mol/L MgCl_2_ (24 U/mg protein), thereafter activity declined and no activity was detected in the presence of 0.5 mol/L MgCl_2_. Obviously, the nature of the cation is not important for MtlD stimulation.

After it had been established that MtlD requires rather high salt concentrations for activity, we tested whether non‐ionic compounds could substitute for salt. Glucose, sucrose, fructose or trehalose (up to 1 mol/L) did not stimulate activity. Glycine betaine taken up from the environment usually represses biosynthesis of compatible solutes, but neither glycine betaine nor choline or mannitol inhibited fructose‐6‐phosphate‐dependent NADPH oxidation catalyzed by MtlD.

Previously it had been surmised that MtlD of *A. baylyi* specifically requires chloride ions (Sand et al., 2013). To analyze a potential chloride stimulation of MtlD from *A. baumannii*, the total salt concentration was kept constant by appropriate addition of another sodium salt. When the total salt concentration was kept constant at 0.5 mol/L by appropriate addition of sodium gluconate, chloride stimulated fructose‐6‐phosphate‐dependent NADPH oxidation by 530% (Figure 5c). A maximum was observed at 400 mmol/L chloride. The same stimulation was observed when NaNO_3_ or Na_2_SO_4_ were used to keep the salt concentration constant (data not shown). At a higher total salt concentration of 1 mol/L, chloride also stimulated activity: sixfold when NaNO_3_ was used to counterbalance or twofold when Na_2_SO_4_ was used to keep the salt concentration constant. These data indicate that MtlD is stimulated by increasing chloride concentrations. However, it has to be mentioned in this context that sodium glutamate had an even more stimulating effect on the enzyme from *A. baumannii,* as the highest activity measured with sodium glutamate (at 1 mol/L) was 200% of the maximal activity detected with NaCl (0.6 mol/L). Glutamate is more likely the physiologically active anion than chloride (see discussion).

### 3.6 Expression of *mtlD* is strictly salt dependent

**Figure 6:**
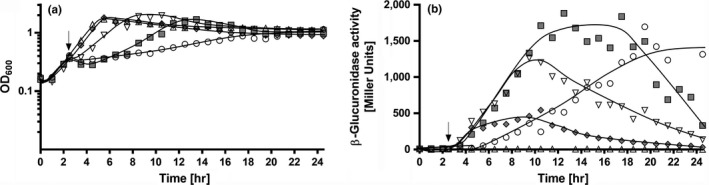
Salt dependence of the *mtlD* promoter activity. Shown are the growth curves of *A. baumannii* transformed with pBAV1k_mtlD‐up‐gusA in mineral medium (a) and the corresponding expression level of the β‐glucuronidase (Miller Units) (b). 200 mmol/L [◊, grey], 300 mmol/L [▽], 400 mmol/L [□, grey], 500 mmol/L [○] NaCl or water [Δ] was added at the timepoint indicated by the arrow. One representative experiment out of at least three independent biological replicates is shown

**Figure 7:**
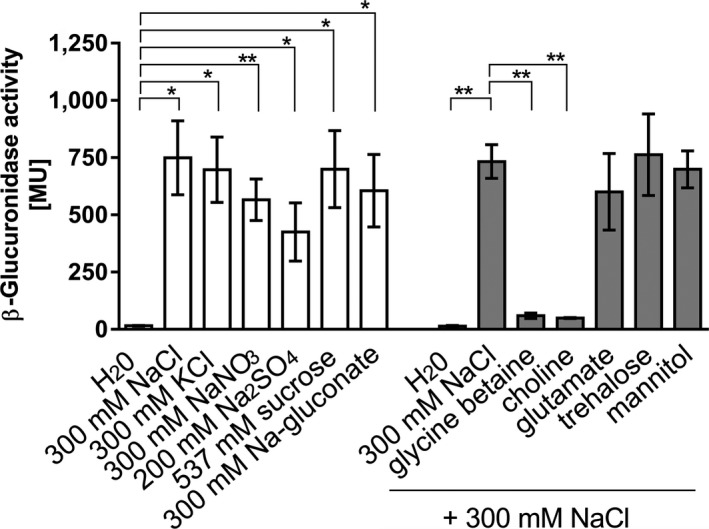
Osmolarity dependence of the *mtlD* promotor activity. Cells were grown in mineral medium with NaCl, KCl, NaNO_3_, Na_2_ SO_4_, sucrose or sodium gluconate as indicated. The osmotic active substances were added in the exponential phase (OD_600_ 0.4‐0.5). Miller Units were calculated 6 hr after the supplementation with the osmolytes (white bars). In another set of experiments, compatible solutes (glycine betaine, choline, glutamate, trehalose or mannitol; 1 mmol/L each) were present in the growth medium and 300 mmol/L NaCl was added in the exponential phase (OD_600_ 0.4‐0.5). Miller Units were calculated 6 hr after the supplementation with NaCl (grey bars). Standard deviation of three biological replicates is given as error bars. Indicated statistical significance by unpaired *t* test *p < .05; **p < .01

To analyze regulation of expression of *mtlD,* a reporter gene assay was used. Therefore, a 669 bp DNA fragment preceding the *mtlD* gene was fused to a promoterless β‐glucuronidase gene in plasmid pIM1440. *A. baumannii* ATCC 19606^T^ was transformed with the plasmid and activation of the *mtlD* promoter was studied using β‐glucuronidase as reporter enzyme. NaCl activated the *mtlD* promoter very strongly when cells were subjected to an osmotic upshock (Figure 6). Activation was concentration dependent with a maximum observed at around 400 mmol/L NaCl. NaCl could be substituted by sucrose, KCl, sodium gluconate, NaNO_3_ or Na_2_SO_4_ (Figure 7). The presence of 1 mM glycine betaine repressed the NaCl‐dependent activity of the *mtlD* promoter (Figure 7). Same was observed for choline, but not for supplied glutamate, trehalose, or mannitol. This is consistent with the observation that trehalose and mannitol are not taken up by *A. baumannii*: they do neither serve as carbon source nor do they improve growth when added to medium with 500 mmol/L NaCl (data not shown). Taken together, these data clearly demonstrate that transcription of *mtlD* is activated by increasing osmolarities/decreasing water activities in the medium.

## 4 DISCUSSION

The accumulation of compatible solutes is a strategy to combat stress caused by hyperosmolarity in the environment (Kempf & Bremer, 1998; Roeßler & Müller, 2001). It is a common response to high salinities that is conserved in all three domains of life, and also *A. baumannii* has been shown to synthesize compatible solutes de novo in the absence of glycine betaine or its precursor (Zeidler et al., 2017). One of the main solutes accumulated by biosynthesis is mannitol, which is a polyol widespread in nature, but rarely used as a compatible solute in bacteria. One example is *A. baylyi*, a nonpathogenic soil organism and close relative of *A. baumannii* (Sand et al., 2013). As *A. baylyi, A. baumannii* uses an unusual bifunctional mannitol‐1‐phosphate dehydrogenase/phosphatase for mannitol biosynthesis (Sand et al., 2015). Deletion of *mtlD* resulted in a loss of mannitol accumulation at high salt. This is clear evidence that *mtlD* is the key gene for mannitol biosynthesis. However, the growth phenotype of the Δ*mtlD* mutant in the presence of high salt is in sharp contrast to the phenotype of the Δ*mtlD* mutant of *A. baylyi*. There, growth was only marginally affected at high salt (lag phase increased by 1.5 hr at 500 mmol/L salt) (Sand et al., 2013). It seems that *A. baumannii* cannot easily compensate for the loss of mannitol by increasing the production of another solute, as for example, glutamate. This is consistent with the finding in *G. oxydans*, where a mutant defective in mannitol production was inhibited in growth at high osmolarities (Zahid & Deppenmeier, 2016). *G. oxydans* can, in addition, take up mannitol from the medium and exogenous mannitol restores growth of the mannitol biosynthesis mutant. *A. baumannii* is not protected by the addition of mannitol to a medium of high osmolarity (data not shown), indicating that mannitol can not be taken up. However, growth of the *mtlD* mutant is restored by the addition of 1 mmol/L glycine betaine to the medium. This indicates that glycine betaine can substitute mannitol and that deletion of *mtlD* affects osmoregulation.

MtlD catalyzes the oxidation of fructose‐6‐phosphate and, like in *A. baylyi*, NADPH is preferred over NADH as the reductant (Sand et al., 2013). The K_M_ value for fructose‐6‐phosphate is 55 mmol/L which is even higher than in *A. baylyi* (14 mmol/L). This is also quite high when compared to other mannitol dehydrogenases, as in the fungus *Aspergillus niger* with a K_M_ of 0.54 mmol/L for fructose‐6‐phosphate (Kiser & Niehaus, 1981) or in brown algae, where mannitol is the main product of photosynthesis (0.28 mmol/L) (Ikawa, Watanabe, & Nisizawa, 1972), but there are also examples where the K_M_ is in the range of *Acinetobacter*, for example mannitol dehydrogenase from *Lactobacillus brevis* with a K_M_ of 70 mmol/L for fructose (Martinez, Barker, & Horecker, 1963). Specific activity of MtlD from *A. baumannii* was only ca. 20% compared to *A. baylyi*, which corresponds to the lower mannitol content in *A. baumannii* (Zeidler et al., 2017).

The most intriguing property of MtlD is its complete inactivity in the absence of salts. The complete lack of enzymatic activity in the absence of salt is quite unusual for a non‐halophile. In the red alga *Caloglossa continua*, mannitol dehydrogenase is also regulated by salt, but it is not inactive without salt, and comparably small concentrations of 100–200 mmol/L only double activity (Iwamoto, Kawanobe, Ikawa, & Shiraiwa, 2003). Addition of different salts fully activates MtlD and the effect is ionic since a mere increase in osmolarity by addition of different sugars did not activate. Since cations and anions are not known to be involved in the reaction mechanism, it is obvious that MtlD senses the ionic composition of the cytoplasm and responds to it by adjusting enzymatic activity. Thus, MtlD is a sensor as well as a catalyst. Since bacteria tend to expel Na^+^ from their cytoplasm and non‐halophiles also Cl^−^, neither Na^+^ nor Cl^−^ seem to be the physiological signal. The same is true for gluconate, nitrate or sulfate. The most likely candidates for activating MtlD under physiological conditions are K^+^ or glutamate. There is no information with respect to the intracellular ion concentrations in *Acinetobacter*, but accumulation of K^+^ and glutamate as counter ion in concentrations up to 400 mmol/L is usually the first response of bacteria to an osmotic upshock (Sleator & Hill, 2002; Tempest, Meers, & Brown, 1970). Many examples are known where K^+^ stimulates the second response, that is, the accumulation of neutral osmoprotectants. In *E. coli*, K^+^ stimulates activity of both the trehalose‐phosphate synthase (Giæver, Styrvold, Kaasen, & Strøm, 1988) and the glutamate dehydrogenase, which is up to 10‐fold more active in the presence of 500 mmol/L potassium (Measures, 1975). Therefore, it is not surprising that K^+^ is considered as a general second messenger for activating enzymes and genes in response to extracellular osmolarity (Booth & Higgins, 1990; Epstein, 2003; Higgins, Cairney, Stirling, Sutherland, & Booth, 1987; Lee & Gralla, 2004). As glutamate is usually accumulated together with potassium ions and since we could show its presence in *A. baumannii*, this amino acid is another possible candidate for being a second messenger.

Many biosynthesis routes for compatible solutes are also controlled at the transcriptional level. Our reporter gene assays revealed that this is also the case for *mtlD* of *A. baumannii*. Nearly no β‐glucuronidase activity was measured in bacteria cultivated in medium without additional salt, but with increasing NaCl concentrations it increased up to 250‐fold. Extreme stimulations are common in the context of compatible solutes. High upregulation is observed for the transporter ProU (>100‐fold) (Cairney, Booth, & Higgins, 1985), and many genes involved in biosynthesis of solutes are stimulated 20‐50‐fold, as for example in the case of ectoine in *H. halophilus* (Saum & Müller, 2008). *mtlD* transcript levels in *A. baylyi* were only 15 times higher in the presence of 500 mmol/L NaCl (Sand et al., 2013). However, one must keep in mind that the reporter gene assays in this study were performed *in trans*, so that the activity measured depends on the plasmid copy number inside the cells.

The *mtlD* promoter was not only activated by addition of salts such as NaCl, KCl or sodium gluconate to the medium, but also by the sugar sucrose. This indicates that, in contrast to regulation at the enzymatic level, low water activity in general is sensed, leading to transcriptional regulation. As mentioned above, the signal could again be glutamate or K^+^, which may be accumulated in response to low water activities independent of the kind of osmolyte present.

In summary, our results identified MtlD as key enzyme in mannitol biosynthesis in *A. baumannii* and unraveled its regulation at the activity level and the transcriptional level. *mtlD* is ubiquitous in *A. baumannii* strains, including AYE, ACICU and other clinical isolates, but whether or not mannitol is also involved in pathobiology of *A. baumannii*, as is the compatible solute trehalose (Gebhardt et al., 2015; Zeidler et al., 2017), remains elusive. Further research is needed to get a deeper understanding of the connection between osmotic stress‐related genes and virulence in this bacterium. The knowledge of the regulation mechanisms involved, as provided by this work, may help in the future to find potential inhibitors of this important nosocomial pathogen.

## CONFLICT OF INTEREST

None declared.

## References

[mbo3614-bib-0001] Averhoff, B. (2015). *Acinetobacter baumannii* ‐ understanding and fighting a new emerging pathogen. Environmental Microbiology Reports, 7, 6–8. 10.1111/1758-2229.12224 25721589

[mbo3614-bib-0002] Baumann, P. , Doudorof, M. , & Stanier, R. Y. (1968). A study of the *Moraxella* group ‐ II. Oxidative‐negative species (genus *Acinetobacter*). Journal of Bacteriology, 95, 1520–1541. 5650064 10.1128/jb.95.5.1520-1541.1968 PMC252171

[mbo3614-bib-0003] Bertani, G. (1951). Studies on lysogenesis. 1. The mode of phage liberation by lysogenic *Escherichia coli* . Journal of Bacteriology, 62, 293–300. 14888646 10.1128/jb.62.3.293-300.1951 PMC386127

[mbo3614-bib-0004] Bligh, E. G. , & Dyer, W. J. (1959). A rapid method of total lipid extraction and purification. Canadian Journal of Biochemistry and Physiology, 37, 911–917. 10.1139/y59-099 13671378

[mbo3614-bib-0005] Boll, J. M. , Tucker, A. T. , Klein, D. R. , Beltran, A. M. , Brodbelt, J. S. , Davies, B. W. , & Trent, M. S. (2015). Reinforcing lipid A acylation on the cell surface of *Acinetobacter baumannii* promotes cationic antimicrobial peptide resistance and desiccation survival. MBio, 6, e00478–00415. 25991684 10.1128/mBio.00478-15 PMC4442142

[mbo3614-bib-0006] Booth, I. R. , & Higgins, C. F. (1990). Enteric bacteria and osmotic stress: Intracellular potassium glutamate as a secondary signal of osmotic stress? FEMS Microbiology Letters, 75, 239–246. 10.1111/j.1574-6968.1990.tb04097.x 1974769

[mbo3614-bib-0007] Bradford, M. M. (1976). A rapid and sensitive method for the quantitation of microgram quantities of protein utilizing the principle of protein‐dye binding. Analytical Biochemistry, 72, 248–254. 10.1016/0003-2697(76)90527-3 942051

[mbo3614-bib-0008] Cairney, J. , Booth, I. R. , & Higgins, C. F. (1985). Osmoregulation of gene expression in *Salmonella typhimurium* ‐ *proU* encodes an osmotically induced betaine transport system. Journal of Bacteriology, 164, 1224–1232. 3905768 10.1128/jb.164.3.1224-1232.1985 PMC219319

[mbo3614-bib-0009] Chaturvedi, V. , Wong, B. , & Newman, S. L. (1996). Oxidative killing of *Cryptococcus neoformans* by human neutrophils ‐ evidence that fungal mannitol protects by scavenging reactive oxygen intermediates. The Journal of Immunology, 156, 3836–3840. 8621921

[mbo3614-bib-0010] Dijkshoorn, L. , Nemec, A. , & Seifert, H. (2007). An increasing threat in hospitals: Multidrug‐resistant *Acinetobacter baumannii* . Nature Reviews Microbiology, 5, 939–951. 10.1038/nrmicro1789 18007677

[mbo3614-bib-0011] Epstein, W. (2003). The roles and regulation of potassium in bacteria. Progress in Nucleic Acid Research and Molecular Biology, 75, 293–320. 10.1016/S0079-6603(03)75008-9 14604015

[mbo3614-bib-0012] Espinal, P. , Martí, S. , & Vila, J. (2012). Effect of biofilm formation on the survival of *Acinetobacter baumannii* on dry surfaces. Journal of Hospital Infection, 80, 56–60. 10.1016/j.jhin.2011.08.013 21975219

[mbo3614-bib-0013] Galinski, E. A. , & Herzog, R. M. (1990). The role of trehalose as a substitute for nitrogen‐containing compatible solutes (*Ectothiorhodospira halochloris*). Archives of Microbiology, 153, 607–613. 10.1007/BF00245273

[mbo3614-bib-0014] Gayoso, C. M. , Mateos, J. , Méndez, J. A. , Fernández‐Puente, P. , Rumbo, C. , Tomás, M. , … Bou, G . (2014). Molecular mechanisms involved in the response to desiccation stress and persistence in *Acinetobacter baumannii* . Journal of Proteome Research, 13, 460–476. 10.1021/pr400603f 24299215

[mbo3614-bib-0015] Gebhardt, M. J. , Gallagher, L. A. , Jacobson, R. K. , Usacheva, E. A. , Peterson, L. R. , Zurawski, D. V. , & Shuman, H. A . (2015) Joint transcriptional control of virulence and resistance to antibiotic and environmental stress in *Acinetobacter baumannii* . MBio, 6, e01660–01615. 26556274 10.1128/mBio.01660-15 PMC4659468

[mbo3614-bib-0016] Giæver, H. M. , Styrvold, O. B. , Kaasen, I. , & Strøm, A. R. (1988). Biochemical and genetic characterization of osmoregulatory trehalose synthesis in *Escherichia coli* . Journal of Bacteriology, 170, 2841–2849. 10.1128/jb.170.6.2841-2849.1988 3131312 PMC211211

[mbo3614-bib-0017] Higgins, C. F. , Cairney, J. , Stirling, D. A. , Sutherland, L. , & Booth, I. R. (1987). Osmotic regulation of gene‐expression: Ionic strength as an intracellular signal? Trends in Biochemical Sciences, 12, 339–344. 10.1016/0968-0004(87)90158-7

[mbo3614-bib-0018] Ikawa, T. , Watanabe, T. , & Nisizawa, K. (1972). Enzymes involved in last steps of biosynthesis of mannitol in brown algae. Plant and Cell Physiology, 13, 1017–1029.

[mbo3614-bib-0019] Iwamoto, K. , Kawanobe, H. , Ikawa, T. , & Shiraiwa, Y. (2003). Characterization of salt‐regulated mannitol‐1‐phosphate dehydrogenase in the red alga *Caloglossa continua* . Plant Physiology, 133, 893–900. 10.1104/pp.103.026906 12972650 PMC219062

[mbo3614-bib-0020] Jawad, A. , Heritage, J. , Snelling, A. M. , Gascoyne‐Binzi, D. M. , & Hawkey, P. M. (1996). Influence of relative humidity and suspending menstrua on survival of *Acinetobacter* spp. on dry surfaces. Journal of Clinical Microbiology, 34, 2881–2887. 8940416 10.1128/jcm.34.12.2881-2887.1996 PMC229427

[mbo3614-bib-0022] Kempf, B. , & Bremer, E. (1998). Uptake and synthesis of compatible solutes as microbial stress responses to high‐osmolality environments. Archives of Microbiology, 170, 319–330. 10.1007/s002030050649 9818351

[mbo3614-bib-0023] Kets, E. P. W. , Galinski, E. A. , de Wit, M. , de Bont, J. A. M. , & Heipieper, H. J. (1996). Mannitol, a novel bacterial compatible solute in *Pseudomonas putida* S12. Journal of Bacteriology, 178, 6665–6670. 10.1128/jb.178.23.6665-6670.1996 8955280 PMC178559

[mbo3614-bib-0024] Kiser, R. C. , & Niehaus, W. G. (1981). Purification and kinetic characterization of mannitol‐1‐phosphate dehydrogenase from *Aspergillus niger* . Archives of Biochemistry and Biophysics, 211, 613–621. 10.1016/0003-9861(81)90496-3 7030216

[mbo3614-bib-0025] Laemmli, U. K. (1970). Cleavage of structural proteins during assembly of head of bacteriophage T4. Nature, 227, 680–685. 10.1038/227680a0 5432063

[mbo3614-bib-0026] Lee, S. J. , & Gralla, J. D. (2004). Osmo‐regulation of bacterial transcription via poised RNA polymerase. Molecular Cell, 14, 153–162. 10.1016/S1097-2765(04)00202-3 15099515

[mbo3614-bib-0027] Lee, C. R. , Lee, J. H. , Park, M. , Park, K. S. , Bae, I. K. , Kim, Y. B. , … Lee, S. H. . (2017). Biology of *Acinetobacter baumannii*: Pathogenesis, antibiotic resistance mechanisms, and prospective treatment options. Frontiers in Cellular and Infection Microbiology, 7, 55. 28348979 10.3389/fcimb.2017.00055 PMC5346588

[mbo3614-bib-0028] Martinez, G. , Barker, H. A. , & Horecker, B. L. (1963). A specific mannitol dehydrogenase from *Lactobacillus brevis* . Journal of Biological Chemistry, 238, 1598–1603.

[mbo3614-bib-0029] Measures, J. C. (1975). Role of amino acids in osmoregulation of non‐halophilic bacteria. Nature, 257, 398–400. 10.1038/257398a0 241020

[mbo3614-bib-0030] Miller, J . (1972). Experiments in molecular genetics. Cold Spring Harbor, NY, Cold Spring Harbor Laboratory.

[mbo3614-bib-0031] Murin, C. D. , Segal, K. , Bryksin, A. , & Matsumura, I. (2012). Expression vectors for *Acinetobacter baylyi* ADP1. Applied and Environment Microbiology, 78, 280–283. 10.1128/AEM.05597-11 PMC3255645 22020504

[mbo3614-bib-0032] Oren, A. (1999). Bioenergetic aspects of halophilism. Microbiology and Molecular Biology Reviews, 63, 334–348. 10357854 10.1128/mmbr.63.2.334-348.1999 PMC98969

[mbo3614-bib-0033] Peleg, A. Y. , de Breij, A. , Adams, M. D. , Cerqueira, G. M. , Mocali, S. , Galardini, M. , … Seifert, H. . (2012). The success of *Acinetobacter* species; genetic, metabolic and virulence attributes. PLoS ONE, 7, e46984 10.1371/journal.pone.0046984 23144699 PMC3483291

[mbo3614-bib-0034] Roca, I. , Espinal, P. , Vila‐Farrés, X. , & Vila, J. (2012). The *Acinetobacter baumannii* oxymoron: Commensal hospital dweller turned pan‐drug‐resistant menace. Frontiers in Microbiology, 3, 148. 22536199 10.3389/fmicb.2012.00148 PMC3333477

[mbo3614-bib-0035] Roeßler, M. , & Müller, V. (2001). Osmoadaptation in bacteria and archaea: Common principles and differences. Environmental Microbiology, 3, 743–754. 10.1046/j.1462-2920.2001.00252.x 11846768

[mbo3614-bib-0036] Sand, M. , de Berardinis, V. , Mingote, A. , Santos, H. , Göttig, S. , Müller, V. , & Averhoff, B. (2011). Salt adaptation in *Acinetobacter baylyi*: Identification and characterization of a secondary glycine betaine transporter. Archives of Microbiology, 193, 723–730. 10.1007/s00203-011-0713-x 21567174

[mbo3614-bib-0037] Sand, M. , Mingote, A. I. , Santos, H. , Müller, V. , & Averhoff, B. (2013). Mannitol, a compatible solute synthesized by *Acinetobacter baylyi* in a two‐step pathway including a salt‐induced and salt‐dependent mannitol‐1‐phosphate dehydrogenase. Environmental Microbiology, 15, 2187–2197. 10.1111/1462-2920.12090 23414076

[mbo3614-bib-0038] Sand, M. , Rodrigues, M. , González, J. M. , de Crécy‐Lagard, V. , Santos, H. , Müller, V. , & Averhoff, B. (2015). Mannitol‐1‐phosphate dehydrogenases/phosphatases: A family of novel bifunctional enzymes for bacterial adaptation to osmotic stress. Environmental Microbiology, 17, 711–719. 10.1111/1462-2920.12503 24800891

[mbo3614-bib-0039] Saum, S. H. , & Müller, V. (2008). Growth phase‐dependent switch in osmolyte strategy in a moderate halophile: Ectoine is a minor osmolyte but major stationary phase solute in *Halobacillus halophilus* . Environmental Microbiology, 10, 716–726. 10.1111/j.1462-2920.2007.01494.x 18093162

[mbo3614-bib-0040] Scholz, A. , Stahl, J. , de Berardinis, V. , Müller, V. , & Averhoff, B. (2015). Osmotic stress response in *Acinetobacter baylyi*: identification of a glycine‐betaine biosynthesis pathway and regulation of osmoadaptive choline uptake and glycine betaine synthesis through a choline‐responsive BetI repressor. Environmental Microbiology, 8, 316–322. 10.1111/1758-2229.12382 26910138

[mbo3614-bib-0041] Sleator, R. D. , & Hill, C. (2002). Bacterial osmoadaptation: The role of osmolytes in bacterial stress and virulence. FEMS Microbiology Reviews, 26, 49–71. 10.1111/j.1574-6976.2002.tb00598.x 12007642

[mbo3614-bib-0042] Stahl, J. , Bergmann, H. , Göttig, S. , Ebersberger, I. , & Averhoff, B. (2015). *Acinetobacter baumannii* virulence is mediated by the concerted action of three phospholipases D. PLoS ONE, 10, e0138360 10.1371/journal.pone.0138360 26379240 PMC4574555

[mbo3614-bib-0043] Stoop, J. M. H. , Williamson, J. D. , & Pharr, D. M. (1996). Mannitol metabolism in plants: A method for coping with stress. Trends in Plant Science, 1, 139–144. 10.1016/S1360-1385(96)80048-3

[mbo3614-bib-0044] Tempest, D. W. , Meers, J. L. , & Brown, C. M. (1970). Influence of environment on content and composition of microbial free amino acid pools. Journal of General Microbiology, 64, 171–185. 10.1099/00221287-64-2-171 4995906

[mbo3614-bib-0045] Tschech, A. , & Pfennig, N. (1984). Growth‐yield increase linked to caffeate reduction in *Acetobacterium woodii* . Archives of Microbiology, 137, 163–167. 10.1007/BF00414460

[mbo3614-bib-0047] Zahid, N. , & Deppenmeier, U. (2016). Role of mannitol dehydrogenases in osmoprotection of *Gluconobacter oxydans* . Applied Microbiology and Biotechnology, 100, 9967–9978. 10.1007/s00253-016-7680-8 27338577

[mbo3614-bib-0048] Zahid, N. , Schweiger, P. , Galinski, E. , & Deppenmeier, U. (2015). Identification of mannitol as compatible solute in *Gluconobacter oxydans* . Applied Microbiology and Biotechnology, 99, 5511–5521. 10.1007/s00253-015-6626-x 25977208

[mbo3614-bib-0049] Zeidler, S. , Hubloher, J. , Schabacker, K. , Lamosa, P. , Santos, H. , & Müller, V. (2017). Trehalose, a temperature‐ and salt‐induced solute with implications in pathobiology of *Acinetobacter baumannii* . Environmental Microbiology, 19, 5088–5099. 10.1111/1462-2920.13987 29124876

[mbo3614-bib-0050] Zhang, X. G. , & Bremer, H. (1995). Control of the *Escherichia coli rrnB* P1 promoter strength by ppGpp. Journal of Biological Chemistry, 270, 11181–11189. 10.1074/jbc.270.19.11181 7538113

