# Supplementary material for: Salt induction and activation of MtlD, the key enzyme in the synthesis of the compatible solute mannitol in Acinetobacter baumannii
Source: Microbiologyopen. 2018 Mar 24;7(6):e00614. doi: 10.1002/mbo3.614 (PMC6291793; doi:10.1002/mbo3.614)
Supplement: Supplementary file 1 [file MBO3-7-e00614-s001.pdf]

S. 1. Primers used in the study

**Primer name**	**Sequence 5' → 3'**	**Application**
mtlD_up_fwd	GTTAGCGGCCGCAACAGCTTGAGGATATGCAGAG	Deletion of *mtlD* in ATCC 19606
mtlD_up_rev	GCCGGGATCCCTTTGGACATTAAGTCGAAC	
mtlD_down_fwd	CTGCGGATCCCATCCATATCGAAGATAGCTC	
mtlD_down_rev	GTTGCTGCAGGTACTACGCTTCTACCAAACC	
mtlD_ctr_fwd	GCTGTACCGCAGTCAATCAC	
mtlD_ctr_rev	GTGTCGTTCTGGTGCCAGAAG	
mtlD_fwd	GCCGCATATGGTGCTTATTTTTCATGGAAAACC	MtlD purification
mtlD_rev	ATGGCGCGGCCGCAACATATTGAGTTGATGTAGTTTTG	
mtlD_up_gusA_fwd	GGCGTCTAGAGCCGAAATTTATGAAGGGAAGC	Reporter gene assay
mtlD_up_gusA_rev	CGCGCCATGGGTTTTTACCTCTTTTTTGATCATG	
